# Marijuana Use and Dependence in Chilean Adolescents and Its Association with Family and Peer Marijuana Use

**DOI:** 10.1007/s12529-016-9595-2

**Published:** 2016-10-03

**Authors:** Mónica Lobato, Robbert Sanderman, Esteban Pizarro, Mariët Hagedoorn

**Affiliations:** 1 University Medical Center Groningen, Health Psychology Section, University of Groningen, FA12, P.O. Box 196, 9700 AD Groningen, The Netherlands; 2 Department of Health Psychology, Health & Technology, University of Twente, Enschede, The Netherlands; 3 Research Department, National Service for Prevention and Rehabilitation of Drug and Alcohol Use (SENDA), Agustinas 1235, 6th floor, Santiago, Chile

**Keywords:** Marijuana, Adolescents, Family, Peers, Substance-related disorders, Cross-sectional

## Abstract

**Purpose:**

The purpose of the study is to examine (1) whether family and peer marijuana use are independently related to adolescent marijuana use in Chile, (2) whether family and peer marijuana use are associated with adolescent marijuana dependence in adolescents using marijuana, and (3) whether the adolescent’s age moderates the association between family or peer use and adolescent marijuana use and/or dependence.

**Method:**

This study used data from the National Survey on Drug Use in the General Population in Chile (a cross-sectional observational study), which was conducted in 2008 and 2010 in 4413 adolescents aged 12–19. Adolescents answered questions about their past-year marijuana use and dependence (ICD-10 criteria) and the marijuana use of their family and peers. Logistic regressions were performed while controlling for confounders.

**Results:**

Adolescents who had a family member who used marijuana were five times more likely to use the drug. Adolescents with a close friend who used marijuana were eight times more likely to use marijuana. When adolescents were using marijuana, they were three times more likely to be dependent if they had a family member who used the drug. However, no significant relationship was found between peer use and dependence. No statistically significant interactions were found between family or peer use and age.

**Conclusion:**

Family and peer marijuana use was independently associated with adolescent’s past-year marijuana use; however, only family marijuana use was statistically associated with adolescent’s marijuana dependence.

## Introduction

Worldwide, marijuana is one of the most commonly used illicit drug in adolescents [1]. Due to mental health and social problems related to drug use, many studies have investigated the factors that predict or moderate marijuana use in adolescents [2]. The last report on marijuana use by the World Health Organization [3] states that most of the studies on risk and protective factors have been conducted in a small number of high-income countries. A limited number of studies performed in developing countries suggest that some of these factors also apply in low- and middle-income countries; however, more research is needed to confirm this hypothesis [3–5]. Therefore, we are uncertain of the generalizability of findings to adolescents from different ethnic and cultural backgrounds [4, 6]. The current study examines marijuana use in Chilean adolescents. In 2012 in Chile, approximately 6.7 % of adolescents aged 12 to 18 years had used marijuana in the past year [7]. Furthermore, 63 % of adolescents who were in a drug treatment program in 2014 declared that marijuana was the primary drug they used [8].

Among the risk and protective factors studied, family influences have received significant attention. It has been found that family members can exert considerable influence over an adolescent’s behavior [9–13]. For example, it is more likely that children will use drugs and alcohol if their parents or siblings use drugs and alcohol [10, 11, 14, 15]. According to behavioral theories, parents or significant others at home may serve as a role model for adolescents. For example, adolescents may imitate their parents and use drugs as a way to cope with their problems [15]. Furthermore, access to substances at home, positive parental norms, and parental perceptions regarding substance use stimulate drug use in adolescents [13]. Moreover, parental drug use could also interfere with important parenting strategies (i.e., adolescent monitoring) that have been shown to be protective against adolescent drug use [13]. Finally, research has shown that some genes may underlie heritable vulnerability to drug dependence [12].

Likewise, peers can be an important source of influence over behavior in adolescents. A systematic review showed that peer use is one of the most important factors related to adolescent marijuana use [5]. There are a number of mechanisms that mediate this influence, including processes of imitation and social learning when they affiliate to peers who use substances [16]. An alternative explanation is peer selection, where adolescents who use drugs select friends with a higher predisposition to use drugs [5].

A few studies have simultaneously examined the drug use of both family members and peers as risk factors for adolescent drug use. Among these, one study found that parental alcohol and marijuana use were not significantly associated with drug use and related psychiatric disorders in adolescents, but the use of marijuana and other drugs by siblings and peers were significant influential factors [17]. Another longitudinal study assessing factors related to marijuana use found that both a familial history of substance use disorder and drug intake by peers were relevant predictors for adolescent marijuana use, with respect to both the initiation and progression of marijuana use [18].

Family and peer influence may be related to age. According to developmental theory, young people entering adolescence undergo a period of heightened susceptibility to family and peer influence, but this tends to diminish when they reach maturity and develop greater personal autonomy in young adulthood [16]. However, there is some controversy about age as a moderator. For example, it has been shown that peers who display deviant behavior such as substance use and/or antisocial behavior had a greater influence on participants’ marijuana abuse at younger ages (14–15 years) compared with older ages (20–21 years) [16]. In contrast, another study related to marijuana use did not find any significant interactions between peer or family drug use and adolescents’ age [18]. Nevertheless, the literature on this topic is scarce and more research is needed.

The current study examines associations between family and peer marijuana use and adolescents’ marijuana use in Chile. It is important to consider some cultural issues with respect to the family. Chilean elementary families (i.e., family members who share a household) often do not consist of only the biological parents and children but have a more diverse composition including members of the extended family (e.g., grandparents and uncles/aunts) and non-relatives who fulfill important family roles [19–22]. Therefore, we used a broader definition of family that includes these individuals. Additionally, studies have shown that Latino immigrant families rate higher than American families in familism, a cultural value that refers to strong family ties, support, and loyalty [23]. This may suggest that the role of the family may be even more influential in Chilean adolescents when compared to Caucasian adolescents. Similarly, high levels of familism might imply that the influence of family on Chilean adolescent drug use is stronger than the influence of peers.

In contrast to most studies, the current study focuses not only on whether adolescents use marijuana but also distinguishes between marijuana use with and without dependence. It is important to understand whether family and peer use are associated with dependence among users. This knowledge can be helpful to develop an effective prevention strategy based on the individual’s profile, such as universal, selective (i.e., preventing use in specific groups), or indicated prevention (i.e., preventing harmful use in users).

In summary, this study tests the following hypotheses: (1) the use of marijuana within the family or by peers will increase the probability of marijuana use in Chilean adolescents, (2) the use of marijuana within the family or by peers will increase the probability of developing dependence in Chilean adolescents using marijuana, and (3) the use of marijuana by family or peers will increase the probability of marijuana use and/or developing dependence, particularly in younger Chilean adolescents.

## Methods

### Participants

This paper presents data from the National Survey on Drug Use in the General Population conducted by the Chilean government, which is a cross-sectional study that has been repeated every 2 years since 1994. The survey is based on structured face-to-face interviews at the participant’s home. Until 2008, a paper version of the questionnaire was used. Since 2010, the questionnaire has been answered using an electronic device, which is designed to provide a private and confidential interview response mode (similar to a self-report questionnaire).

Each time the survey is conducted, the National Institute of Statistics selects a sample to be a representative of the Chilean population between 12 and 65 years old and includes both genders and all socio-economic statuses. The sample is selected following a three-stage sampling procedure. In stage 1, city blocks are randomly selected from all cities to represent at least 70 % of the total of urban areas for each region. In stage 2, addresses within these city blocks are randomly selected. Finally, in stage 3, when the fieldwork takes place, the interviewer randomly selects one person at each of the selected addresses.

The current analysis is based on 4413 adolescents. Following the World Health Organization’s definition of adolescence and to test hypothesis 3, the study includes the full age range of adolescence from 12 to 19 years. Moreover, this study includes data from the years 2008 and 2010.

### Measures

#### Adolescent Marijuana Use

##### Adolescent past-year marijuana use

Participants who gave a positive answer to the question, “Have you ever tried marijuana?”, were then asked to indicate the last time they used marijuana, with possible answers ranging from (1) in the last 30 days, (2) more than 30 days but less than 12 months ago, and (3) more than 12 months ago. Based on this question, participants who answered (1) or (2) met the criterion for past-year marijuana use.

##### Adolescent marijuana dependence

Dependence was measured based on the six symptoms described by the International Statistical Classification of Diseases and Related Health Problems (ICD)-10 for drug dependence [24]. Four of these symptoms were assessed with two questions each. For example, to assess whether their control is impaired, the participants were asked, “Have you used marijuana even though you intended not to do so?” and “Have you ever ended up smoking marijuana a lot more than you intended?” Participants providing a positive answer to either question were categorized as showing impaired control. Adolescents with three or more symptoms were categorized as “dependent users,” adolescents with less than three symptoms were categorized as “non-dependent users,” and adolescents who did not use in the preceding year were categorized as “non-users.”

#### Family and Peer Marijuana Use

##### Family marijuana use

Participants answered (yes/no) to the question, “At home, as far as you know, does anyone use marijuana? (If you use marijuana, do not count yourself in your answer).” People with whom the adolescent lives were considered part of his or her family, which is in accordance with the broader definition of family described above. Additionally, when selecting the person to be interviewed, it was identified if there were more than one family living within the same household (INE, 2011); the question was answered with respect to their own family only.

##### Peer marijuana use

This variable was evaluated based on the answer (yes/no) to the question, “As far as you know, do you have any close friends that use marijuana?”

#### Background Variables

##### Demographic variables

Socioeconomic status was assessed by the trained interviewer based on two questions that could be answered using a list of objective characteristics: “What is the quality of the neighborhood?” (1 = “residential neighborhood on an expensive terrain” to 5 = “neighborhood unpaved, dirty and/or unhealthy and/or unsanitary conditions”) and “What is the quality of the dwelling where the interview was conducted and the interviewee lives?” (1 = “luxurious and big house/apartment” to 5 = “wooden house without drinking water and/or sewerage”). These two questions were derived from the Graffar instrument (Méndez and Méndez, 1994), a validated scale created to assess SES for research purposes. The scores of the two questions were summed and categorized into low [10–9], middle low [8–7], middle [6], middle high [5–4], and high [3–2]. For the present research, the low and middle low [10–7] categories and the high and middle high [5–2] categories were merged, due to the limited number of cases within the extreme categories. Occupational status was measured using the following categories: studying, working, or at home (neither studying nor working).

##### Adolescent past-year alcohol use

This variable was assessed similarly to adolescent past-year marijuana use.

##### Neighborhood risk

This variable was assessed with seven questions related to the perception of the neighborhood where the participant lives. The main question was, “As far as you know, do the following things occur in your neighborhood?” and was accompanied by a list of situations such as “drug trafficking” and “house burglaries.” Answers were given on a Likert scale (0 as never to 4 as too much). The items were summed into a single scale with a higher score indicating higher neighborhood risk (Cronbach’s alpha = 0.87). Missing total scores (*N* = 247; 5.6 %) were imputed using the mean of the smallest demographic division included in both survey years (municipality). A sensitivity analysis was conducted to test the robustness of the results. This sensitivity analyses showed that the results were similar, with or without imputation. Therefore, the imputed variables were included to keep as many cases as possible in the regression analyses.

##### Survey year

This variable was included to account for variability in prevalence of marijuana use in each survey year.

### Data Analysis

Data management and analyses were performed using SPSS 22.0 (2013). For each variable, missing values were less than 2 %. Thirteen cases were eliminated from the original sample because of the inconsistency between the two outcome variables. Specifically, nine participants declared having used marijuana in the past 12 months but did not answer the questions related to marijuana dependence. Additionally, four cases indicated no marijuana use in the last 12 months but answered the dependency section.

The literature indicates that social environment (i.e., neighborhood), socioeconomic status, and alcohol use are associated with marijuana use [5, 17, 18, 25–30], in addition to gender and age. In addition, adolescent’s occupational status has been shown to be related to drug use in adolescents in the Chilean population [31, 32]. Accordingly, these variables were included as confounders.

A binary logistic regression analysis and a multinomial logistic regression analysis with past-year marijuana use and marijuana dependence as dependent variables, respectively, were performed to test the hypotheses, after controlling for potential confounders. Furthermore, two interaction variables were added to the two models to test age as a potential moderator of the predicted associations between family and peer marijuana use and adolescent marijuana use.

## Results

### Sample Characteristics

Table 1 presents the summary statistics for the sample. In our study, 51 % of the sample was male with a mean age of 15.7 years (SD = 2.3). Only 7 % (*n* = 305) reported having used marijuana in the past year and a small group met the ICD-10 criteria for dependence (*n* = 74; 2 %). Approximately, one-third of the sample declared having a close friend that used marijuana (*n* = 1206; 28 %), and 5 % (*n* = 198) stated that someone in their family was using marijuana.

**Table 1:** Summary statistics. Number, percentage, mean, and standard deviation

	n (%)	M (SD)	Missing values *n* (%)
Sample size	4413 (100)		
Age (range: 12–19)	–	15.7 (2.3)	–
Gender (male)	2253 (51.1)	–	–
Occupational status	–	–	24 (0.5)
Studying	3673 (83.7)	–	
Working	337 (7.7)	–	
At home	379 (8.6)	–	
Socioeconomic status	–	–	27 (0.6)
Low	1292 (29.5)	–	
Middle	1834 (41.8)	–	
High	1260 (28.7)	–	
Adolescent year alcohol prevalence (yes)	1741 (39.5)	–	–
Neighborhood risk (range: 0–28)	–	13.6 (5.6)	–
Survey year (2008)	2119 (48.0)		–
Peer Marijuana use (yes)	1206 (27.7)	–	66 (1.5)
Family Marijuana use (yes)	198 (4.5)	–	37 (0.8)
Adolescent year marijuana prevalence (yes)	305 (6.9)	–	–
Adolescent marijuana dependence	–	–	–
Non–users	4108 (93.1)	–	
Non–dependent users	231 (5.2)	–	
Dependent users	74 (1.7)	–	

*n* number, *%* percentage, *M* mean, *SD* standard deviation

Table 2 shows the means and percentages of the background variables and potential predictors for the different levels of the two outcomes. Adolescents using marijuana in the past year were more likely to be an older male and live with a family member or have a close friend who used marijuana than adolescents who had not used the drug. The largest difference was observed for peer marijuana use (no use = 24 %, use = 83 %). Adolescents who met the criterion for dependence were more likely to be male and have a family member and peers who used marijuana than adolescents who were classified as non-dependent users. The largest difference was observed for family marijuana use (non-dependent user = 19 %, dependent user = 38 %).

**Table 2:** Means and percentages of the background and predictor variables by outcomes

	Adolescent past-year marijuana use		Adolescent Marijuana dependence	
	No	Yes	Non-dependent users	Dependent users
Age (M, SD)	15.6 (2.3)	17.2 (1, 5)	17.2 (1.4)	17.2 (1.5)
Gender				
Male (%, *n*)	50.0 (2055)	64.9 (198)	64.1 (148)	67.6 (50)
Occupational status				
Studying (%, *n*)	85.2 (3479)	63.6 (194)	66.7 (154)	54.1 (40)
Working (%, *n*)	7.0 (286)	16.7 (51)	17.3 (40)	14.9 (11)
At home (%, *n*)	7.8 (319)	19.7 (60)	16.0 (37)	31.1 (23)
Socioeconomic status				
Low (%, *n*)	29.1 (1186)	34.9 (106)	30.4 (70)	48.6 (36)
Middle (%, *n*)	42.0 (1715)	39.1 (119)	40.9 (94)	33.8 (25)
High (%, *n*)	28.9 (1181)	26.0 (79)	28.7 (66)	17.6 (13)
Adolescent year alcohol use				
Yes (%, *n*)	35.4 (1454)	94.1 (287)	93.1 (215)	97.3 (72)
Neighborhood risk (M, SD)	13.5 (5.4)	15.1 (5, 6)	14.8 (5.7)	16.1 (5.4)
Survey year				
2008 (%, *n*)	47.0 (1931)	61.6 (188)	56.7 (131)	77.0 (57)
Peer marijuana use				
Yes (%, *n*)	23.6 (954)	82.9 (252)	81.7 (188)	86.5 (64)
Family marijuana use				
Yes (%, *n*)	3.1 (128)	23.3 (70)	18.5 (42)	38.4 (28)

*n* number, *%* percentage, *M* mean, *SD* standard deviation

### Adolescent Marijuana Use: the Role of Family and Peer Use

Regarding the first hypothesis (the use of marijuana within the family or by peers will increase the probability of marijuana use in Chilean adolescents), the binary logistic regression (Table 3) showed that family marijuana use was significantly positively associated with adolescent past-year marijuana use (*p* < 0.001), after controlling for potential confounders. Consequently, adolescents who had a family member living at home using marijuana were five times more likely to use the drug (OR = 5.19; [3.221, 8.350]). Likewise, the same regression revealed that peer marijuana use was also significantly positively associated with adolescent past-year marijuana use (*p* < 0.001), after controlling for potential confounders. Specifically, adolescents were eight times more likely to use marijuana if they had a close friend who used marijuana (OR = 7.70; [5.007, 11.836]).

**Table 3:** Results of the binary logistic regression analysis for adolescent past-year marijuana use

	Unadjusted			Adjusted		
	*p*	OR (95 % CI)		*p*	OR (95 % CI)	
Constant				<0.001	0.002	
Age				0.060	1.172	(0.993–1.383)
Gender (male)				**<** *0.001*	*1.913*	(*1.437–2.548*)
Occupational status (studying/reference)				0.001	–	–
Occupational status (working)				0.220	1.290	(0.859–1.936)
Occupational status (at home)				*<0.001*	*2.121*	(*1.438–3.128*)
Socioeconomic status (low/reference)				0.915	–	–
Socioeconomic status (middle)				0.836	0.966	(0.697–1.339)
Socioeconomic status (high)				0.674	0.922	(0.633–1.344)
Adolescent past-year alcohol use (yes)				**<** *0.001*	*12.999*	(*7.573–22.311*)
Neighborhood risk				0.372	0.988	(0.964–1.014)
Survey year (2008)				0.322	0.865	
Peer marijuana use (yes)	**<** *0.001*	*15.692*	(*11.541–21.335*)	**<** *0.001*	*7.698*	(*5.007–11.836*)
Family marijuana use (yes)	<*0.001*	*9.387*	(*6.815–12.931*)	<*0.001*	*5.186*	(*3.221–8.350*)
Age × peer marijuana use (yes)				0.311	0.908	(0.754–1.094)
Age × family marijuana use (yes)				0.137	0.846	(0.679–1.055)

Significant *p* values and odd ratios are printed in italics

*n* = 4269

*p p* value, *OR* odds ratio, *CI* confidence interval

Regarding the second hypothesis (the use of marijuana within the family or by peers will increase the probability of developing dependence in Chilean adolescents using marijuana), the multinomial logistic regression (Table 4) revealed that family marijuana use was significantly positively associated with adolescent marijuana dependence (*p* < 0.001) when non-users were used as a reference category. Moreover, the results presented a significant positive relationship for family marijuana use when non-dependent users were compared with dependent users (*p* = 0.015), after controlling for potential confounders. Therefore, if the adolescents who were using marijuana had a family member living at home using the same drug, they were approximately three times more likely to develop dependence (OR = 2.67; [1.210, 5.907]). However, although peer marijuana use showed a trend similar to family use when non-users were used as a reference category, no significant relationship was found when comparing non-dependent users with dependent users (*p* = 0.683), after controlling for potential confounders.

**Table 4:** Results of the multinomial logistic regression analysis for adolescent marijuana dependence

	Reference category non-user							Ref. cat. non-problem user		
	Non-dependent user			Dependent user				Dependent user		
	*p*	OR (95 % CI)		*p*	OR (95 % CI)			*p*	OR (95 % CI)	
Intercept	<0.001	–	–	<0.001	–	–		0.019	–	–
Age	0.112	1.155	(0.967–1.380)	0.251	1.285	(0.838–1.971)		0.649	1.113	(0.703–1.760)
Gender (male)	<*0.001*	*1.747*	(*1.279–2.386*)	*0.001*	*2.736*	(*1.567–4.778*)		0.142	1.566	(0.860–2.850)
Occupational status (studying/reference)	–	–	–	–	–	–		–	–	–
Occupational status (working)	0.227	1.309	(0.846–2.026)	0.569	1.256	(0.574–2.749)		0.922	0.959	(0.418–2.203)
Occupational status (at home)	*0.029*	*1.637*	(*1.051–2.551*)	<*0.001*	*4.417*	(*2.339–8.344*)		*0.005*	*2.698*	(*1.344–5.415*)
Socioeconomic status (low/reference)	–	–	–	–	–	–		–	–	–
Socioeconomic status (middle)	0.644	1.089	(0.758–1.566)	0.208	0.689	(0.385–1.232)		0.154	0.632	(0.336–1.188)
Socioeconomic status (high)	0.767	1.064	(0.705–1.607)	0.126	0.559	(0.265–1.177)		0.112	0.525	(0.237–1.163)
Adolescent past-year alcohol use (yes)	<*0.001*	*11.588*	(*6.496–20.671*)	<*0.001*	*23.293*	(*5.436–99.813*)		0.378	2.010	(0.426–9.490)
Neighborhood risk	0.281	0.985	(0.958–1.013)	0.941	1.002	(0.956–1.050)		0.507	1.017	(0.967–1.070)
Survey year (2008)	0.828	1.035	(0.760–1.410)	*0.004*	*0.400*	(*0.216–0.741*)		*0.004*	*0.387*	(*0.202–0.741*)
Peer marijuana use (yes)	<*0.001*	*7.476*	(*4.694–11.906*)	*<0.001*	*9.518*	(*3.236–27.993*)		0.683	1.273	(0.399–4.059)
Family marijuana use (yes)	<*0.001*	*3.838*	(*2.327–6.838*)	<*0.001*	*10.663*	(*5.073–22.414*)		*0.015*	*2.674*	(*1.210–5.907*)
Age × peer marijuana use (yes)	0.551	0.940	(0.769–1.151)	0.264	0.774	(0.494–1.214)		0.431	0.823	(0.507–1.337)
Age × family marijuana use (yes)	0.140	0.828	(0.644–1.064)	0.438	0.874	(0.621–1.229)		0.776	1.055	(0.730–1.524)

Significant *p* values and odd ratios are printed in italics

*n* = 4269

*p p* value, *OR* odds ratio, *CI* confidence interval

Regarding the third hypothesis (the use of marijuana by family or peers will increase the probability of marijuana use and/or developing dependence, particularly in younger Chilean adolescents), in both logistic regressions, no significant interactions were found between age and either adolescent past-year marijuana use (age and family marijuana use *p* = 0.137; age and peer marijuana use *p* = 0.311) or adolescent marijuana dependence when comparing adolescents using marijuana without dependence and those who met the criterion for dependence (age and family marijuana use *p* = 0.776; age and peer marijuana use *p* = 0.431).

## Discussion

The present study examined the associations among family drug use and peer drug use, and drug use and dependence in Chilean adolescents. To our knowledge, this is the first study that compared not only users and non-users but also adolescents with and without dependence.

Family drug use was significantly associated with adolescent past-year marijuana use after controlling for peer drug use and background variables. This finding is consistent with previous studies conducted among Caucasian populations [9, 12, 13]. This is interesting considering that there are cultural differences regarding the definition and meaning of family. Following the social learning theory, modeling takes place when an individual is valued by the learner. Family members, regardless of whether they are a blood relative or not, are valued based on their role or function within the family and therefore serve as an important role model [11, 15]. In other words, it may be more important to focus on the role of the individual within the family rather than the blood tie of family members when examining their influence on adolescents’ drug use. Unfortunately, our measurement does not allow for the disentanglement of the different types and roles of family members. It would be interesting to examine this in future research.

Also, in line with the literature, peer marijuana use was significantly related to past-year marijuana use in adolescents [5]. Peer marijuana use presented a level of risk similar to that of family marijuana use that has been found in other studies [18]. Peers who use drugs are role models who exert covert (through social learning and imitation processes) and perhaps also overt pressure on their non-using peers to use marijuana [5].

Family drug use was also a significant factor for marijuana dependence, suggesting that family drug use exerts an important influence over adolescents’ behavior and mental health. However, although adolescents were more likely to use marijuana when a close friend was using the drug, peer use had no effect on using the drug without dependence or developing dependence. Unfortunately, there is no literature to compare the results regarding marijuana dependence. Nonetheless, it appears that, in the progression to more severe drug use, family members are more influential than peers. Within this context, the concept of familism might play an important role by covertly pushing the adolescent to copy the drug use of family members if the family sets no clear limits with respect to adolescent drug use. Another possible explanation could be biological mechanisms, where genes associated with drug dependence are heritable to offspring; therefore, children of drug-dependent parents are more likely to develop dependency (i.e., when they start using drugs) than adolescents without genetic vulnerability.

Based on developmental theories, we expected to find a stronger association between adolescent marijuana use and the use of marijuana by peers and/or parents at younger ages, due to the higher susceptibility to social influences at younger ages [16]. Nonetheless, and in line with one other study [18], we did not find any support for such an interaction. One possible explanation could be the rare cases of drug use between 12 and 14 years old (15 out 305). Furthermore, one might wonder whether a two-way interaction (family and peer drug use) or a three-way interaction (including age) may exist. However, additional analyses (results not presented) did not show such interactions.

The current results, which are based on a large national database from a well-known survey that has been conducted every 2 years since 1994, should be interpreted while considering a number of limitations. First, the cross-sectional design of the study does not warrant conclusions regarding causality. Second, although the broader concept of family used in this study does considers the cultural context of the study group and adds value to the literature, it limits the analysis. Specifically, we were unable to differentiate between marijuana use in parents, siblings, and other family members. This could conceal important information because the previous literature suggests a differential influence of parents and siblings [17].

The findings from the present study have potential implications for mental health professionals. Drug prevention programs need to be cognizant of family and peer drug use because they are important factors associated with adolescent marijuana use, despite the age of the adolescent. Due to the association between family drug use and adolescent drug use, it is important to develop programs for children living with a family member in drug treatment, including selective prevention for young children (to prevent drug use) and indicated prevention for adolescents who are non-dependent drug users (to prevent developing dependence). In the case of drug treatment programs for adolescents, professionals should be aware that adolescent who uses marijuana is more likely to develop marijuana dependence if a family member uses marijuana. Therefore, it is necessary to address this issue with the family, possibly by discussing parents’ perceptions, attitudes, and beliefs about their own use and their adolescent’s use.

In conclusion, this study, which is based on a large national sample in Chile, has suggested that family marijuana use is significantly associated with adolescent marijuana use after peer influence was added to the analyses. Peer marijuana use showed a similar pattern to family drug use with regards to past-year marijuana use, but only family marijuana use was significantly associated with marijuana dependence. Age did not moderate the association between adolescent use and family or peer marijuana use. Further research is needed to examine whether the specific family member using marijuana influences adolescent marijuana use and to determine what family factors may help adolescent drug users quit or reduce drug use.
